# Accounting for dropout in xenografted tumour efficacy studies: integrated endpoint analysis, reduced bias and better use of animals

**DOI:** 10.1007/s00280-016-3059-x

**Published:** 2016-05-25

**Authors:** Emma C. Martin, Leon Aarons, James W. T. Yates

**Affiliations:** Centre for Applied Pharmacokinetic Research, Manchester Pharmacy School, The University of Manchester, Stopford Building 3.32, Oxford Road, Manchester, M13 9PT UK; AstraZeneca, Innovative Medicines, Oncology, Modelling and Simulation, Li Ka Shing Centre, Robinson Way, Cambridge, CB2 0RE UK

**Keywords:** Dropout, Joint models, Xenograft, Tumour growth models

## Abstract

**Purpose:**

Xenograft studies are commonly used to assess the efficacy of new compounds and characterise their dose–response relationship. Analysis often involves comparing the final tumour sizes across dose groups. This can cause bias, as often in xenograft studies a tumour burden limit (TBL) is imposed for ethical reasons, leading to the animals with the largest tumours being excluded from the final analysis. This means the average tumour size, particularly in the control group, is underestimated, leading to an underestimate of the treatment effect.

**Methods:**

Four methods to account for dropout due to the TBL are proposed, which use all the available data instead of only final observations: modelling, pattern mixture models, treating dropouts as censored using the M3 method and joint modelling of tumour growth and dropout. The methods were applied to both a simulated data set and a real example.

**Results:**

All four proposed methods led to an improvement in the estimate of treatment effect in the simulated data. The joint modelling method performed most strongly, with the censoring method also providing a good estimate of the treatment effect, but with higher uncertainty. In the real data example, the dose–response estimated using the censoring and joint modelling methods was higher than the very flat curve estimated from average final measurements.

**Conclusions:**

Accounting for dropout using the proposed censoring or joint modelling methods allows the treatment effect to be recovered in studies where it may have been obscured due to dropout caused by the TBL.

**Electronic supplementary material:**

The online version of this article (doi:10.1007/s00280-016-3059-x) contains supplementary material, which is available to authorized users.

## Introduction

Xenograft studies are the most common preclinical studies used to assess the antitumour effects of new compounds and involve grafting human cancer cells into the flanks of immune deficient mice. The main purpose of these studies is to assess the efficacy of the compound and characterise the dose–response relationship [1]. Analysis often involves comparing the final tumour sizes across dose groups to look for evidence of tumour shrinkage, which can be done in a number of ways, such as carrying out a *t* test on the final tumour sizes in each group, calculating a tumour growth index [2], or using adapted RECIST criteria to categorise response [3].

Comparing the tumour sizes from the final day of the study alone can cause potential issues, as any animals that dropout early are excluded from the analysis, which is equivalent to carrying out a complete case analysis, known to cause bias when data are not missing at random [4]. Often in xenograft studies, a tumour burden limit (TBL) is imposed for ethical reasons, as tumour burden should be kept to the minimum required in all xenograft studies [5]. Once an animal’s tumour has reached this limit, it is removed from the study. The limit used varies and, for example, may be when the tumour has quadrupled in size from baseline [1, 6], the mean diameter exceeds 1.2 cm [5] or the weight of the tumour exceeds 2.5 g, as used in the following examples. The limit chosen will depend on the objective of the experiment.

Dropout due to the tumour burden limit means that animals with larger tumours are removed first from the study, reducing the group average tumour size, which disproportionately affects the control group where tumours are expected to be largest. This causes a bias in the estimate of treatment effect, as the tumour size in the control group is being underestimated, and so any tumour shrinkage caused by the compound will look less significant [1, 7]. This is an example of informative dropout, as whether an animal drops out is dependent on unobserved measurements that would have been observed if dropout had not occurred [8, 9], and as such is non-ignorable.

This dropout effect has previously been discussed in the literature, notably by Pan et al. [6], where dropout caused by the tumour burden limit is modelled using a survival model and tumours that are too small to be measured are modelled using a logistic model. Tan et al. [1] also discuss this issue and suggest the use of the expectation/conditional maximisation (ECM) algorithm.

Informative dropout in a wider context has also been extensively investigated. Wu and Carroll [10] focus particularly on informative right censored data as is observed here and find that it can cause substantial bias and reduction in power when comparing group differences. Bjornsson et al. [11] look at the effect of informative dropout on parameter estimation in nonlinear fixed effects models and found that bias of up to 21 % in parameter estimates could be found if dropout was not accounted for in the analysis. Many methods for dealing with informative dropout have been suggested. Simple methods exist, such as last observation carried forwards (LOCF) where all missing measurements are replaced with the last measurement taken, which in the case of xenograft studies would be a highly conservative approach. Other methods such as the use of ECM algorithms [1], selection models [12], pattern mixture models [12] and various methods to jointly model the dropout with the endpoint [13, 14] have also been suggested but most have not been applied to the problem of tumour burden limit.

In the present study, four methods for accounting for dropout due to the tumour burden limit were investigated on both a simulated data set and a real data set to see whether they could improve the estimate of the treatment effect. Throughout this paper, tumour growth has been simulated and modelled using the Simeoni model as the base model [15]; however, the same methods could be applied to other models of tumour growth.

## Materials and methods

### Dropout methods

Four methods proposed for dealing with informative dropout due to the tumour burden limit in xenograft experiments are explained below. Each method results in a model for tumour growth, from which tumour size can be estimated at any time. This allows a comparison between dose groups which is based on all available data, rather than focussing on a single time point. The models can also be used for simulating future preclinical trials and translation to clinical trials.

#### Modelling

The modelling method involves the fitting of a tumour growth model to the available data. Let *f*(*t*) be the structural part of the model, which will give the expected tumour size at time *t*. If *e*(*t*) is the residual error which is assumed to arise from a distribution with mean zero and variance *g*(*t*), the observed tumour size will be given by *y*(*t*) = *f*(*t*) + *e*(*t*).

#### Pattern mixture

The pattern mixture method treats the population as a combination of animals with different patterns of dropout and those who complete the study. The population parameters are then averaged across the patterns [12]. The data are split into dropout patterns based on the time of the final sample, and these dropout patterns can be collapsed to ensure a reasonable amount of data remains in each pattern. Here, the available case missing value (ACMV) method is used; meaning data for each pattern are imputed based on a model fitted to the data for animals that dropped out at a later date. In the current analysis, 5 data sets have been imputed. The same model is then fitted to each of the imputed data sets, and the results averaged to obtain the pooled parameter estimates. The variance of each parameter estimate (*V*_*β*_) can be computed using Eqs. 1 and 2, referred to as Rubin’s rules [16].

1 $$V_{\beta } = \bar{U}_{\beta } + \left( {1 + \frac{1}{M}} \right) \cdot B_{\beta }$$

2 $$\begin{aligned} \bar{U}_{\beta } & = \frac{1}{M} \mathop \sum \limits_{m = 1}^{M} U_{\beta }^{m} ,\quad B_{\beta } = \frac{1}{M - 1} \mathop \sum \limits_{m = 1}^{M} \left( {\hat{\beta }^{m} - \bar{\beta }} \right)^{2} , \\ \beta & = \frac{1}{M} \mathop \sum \limits_{m = 1}^{M} \hat{\beta }^{m} ,\quad U_{\beta }^{m} = {\text{Var}}\left( {\hat{\beta }^{m} } \right) \\ \end{aligned}$$

where $\bar{U}_{\beta }$ is the pooled within-imputation variance, *M* is the number of imputations, *B*_*β*_ is the between-imputation variance of estimates, $\hat{\beta }^{m}$ is the vector of parameter estimates for imputed data set *m*, $\bar{\beta }$ is the pooled parameter estimates and *U*_*β*_^*m*^ is the within-imputation variance of estimates.It is not possible to simulate from a pattern mixture model; therefore, it is not possible to calculate the confidence intervals for the dose–response, as the method cannot be bootstrapped and standard errors cannot be calculated; this means that there will be no estimate of uncertainty around the dose–response and whether the dose groups are significantly different to the control group cannot be determined.

#### Censoring

In the censoring method, the same base model can be used as in the methods above, but the observations missing due to dropout are treated as censored using the M3 method from Beal [17]. This method is often used to model drug concentration data that are below the limit of quantification (BLQ), whilst here it is assumed that the tumour size is above the tumour burden limit. The M3 method simultaneously models the continuous tumour growth data and the planned observations that were missing due to dropout which are treated as categorical. The likelihood for missing values is replaced by the likelihood of the missing value truly being above the tumour burden limit, given that the observation is missing due to dropout.

The likelihood for non-missing observations below the tumour burden limit is calculated using Eq. 3.

3 $$l\left( t \right) = \frac{1}{{\sqrt {2\pi g\left( t \right)} }} \exp \left( { - \frac{1}{2}\left( {\frac{{\left( {y\left( t \right) - f\left( t \right)} \right)^{2} }}{g\left( t \right)}} \right)} \right)$$

The likelihood for those points missing due to being above the TBL is assumed to be the likelihood the observation would truly be above the TBL, as shown in Eq. 4.

4 $$l\left( t \right) = 1 -\Phi \left( {\frac{{{\text{TBL}} - f\left( t \right)}}{{\sqrt {g\left( t \right)} }}} \right)$$

where Φ is the cumulative normal distribution function. This differs from that used when M3 is being used to model BLQ data, as the cumulative density is taken from 1, as we are interested in the probability of being above the limit, unlike for BLQ where we are interested in the probability of being below the limit.

#### Joint modelling

In the joint modelling method, the same base model can be used as in the methods above to describe the tumour growth profiles and then the missing data are modelled using logistic regression. The two models are jointly fitted through the sharing of random effects.

A dropout model similar to that in Hansson et al. [14] is proposed, where it was used to simulate dropout, dependent on the observed sum of longest diameters, progressive disease and time since the start of the study. Here, dropout is assumed to depend on tumour size only, as it does not directly depend on the time in the study, particularly for those in the treated groups where dropout occurred later. The logistic model is fitted to each time point independently using Eqs. 5 and 6.

5 $$x = \theta_{\text{intercept}} + \theta_{\text{tumour size}} + {\text{random effect}}$$

6 $$\Pr_{\text{drop out}} = \frac{\exp \left( x \right)}{1 + \exp \left( x \right)}.$$

### Simulated data

A data set was simulated using a model of the anticancer drug paclitaxel’s effect on tumour growth in xenograft studies reported in Simeoni et al. [15]. The model was derived after the dosing of paclitaxel as an alcoholic solution of Cremophor to animals bearing A2780 tumours. The Simeoni model describes tumour growth with an initial exponential growth phase, followed by a linear growth phase and uses transit compartments to describe the delay in the effect of the drug on tumour size.

**Fig. 1:**
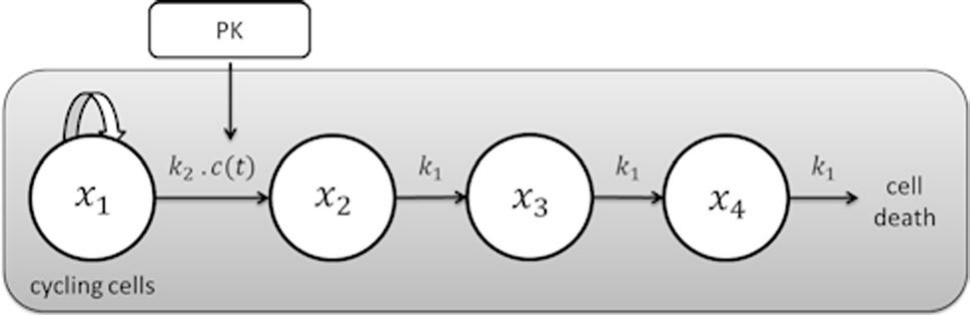
Diagram of the Simeoni model of tumour growth, *C*(*t*) is the concentration of the drug at time *t*, with *K*_2_ estimating its potency. The overall tumour size is the sum of *x*_1_–*x*_4_, and *K*_1_ is the rate constant for the transit of cells. The increase in cycling cells can be described by an exponential growth phase followed by a linear growth phase. This figure has been adapted from one presented in Simeoni et al. [15] 56 × 18 mm (300 × 300 DPI)

The drug concentrations were simulated from the reported two compartment intravenous model, with volume of distribution 0.81 L/kg, *K*_10_ 0.868, *K*_12_ 0.006 and *K*_21_ 0.0838 h^−1^, which was assumed to be the same for all animals, as no variation in parameter values was provided. The tumour growth profiles were simulated using the parameter estimates in the original paper for paclitaxel experiment 1, which can be found in the results table, and with the tumour growth model shown in Fig. 1, and described by Eq. 7. Data were simulated for 72 animals in total, 24 control animals, 24 receiving 4 mg/kg daily and 24 receiving 8 mg/kg daily starting on day 8. The same observation times were used as in the original experiment, but a simpler dosing schedule was chosen to reduce the impact of dosing times on parameter estimation.

7 $$\begin{aligned} \frac{{{\text{d}}x_{1} \left( t \right)}}{{{\text{d}}t}} & = \frac{{\lambda_{0} \cdot x_{1} \left( t \right)}}{{\left[ {1 + \left( {\frac{{\lambda_{0} }}{{\lambda_{1} }} \cdot w\left( t \right)} \right)^{\psi } } \right]^{{{\raise0.7ex\hbox{$1$} \!\mathord{\left/ {\vphantom {1 \psi }}\right.\kern-0pt} \!\lower0.7ex\hbox{$\psi $}}}} }} - k_{2} \cdot c\left( t \right) \cdot x_{1} \left( t \right) \\ \frac{{{\text{d}}x_{2} \left( t \right)}}{{{\text{d}}t}} & = k_{2} \cdot c\left( t \right) \cdot x_{1} \left( t \right) - k_{1} \cdot x_{2} \left( t \right) \\ \frac{{{\text{d}}x_{3} \left( t \right)}}{{{\text{d}}t }} & = k_{1} \cdot \left( {x_{2} \left( t \right) - x_{3} \left( t \right)} \right) \\ \frac{{{\text{d}}x_{4} \left( t \right)}}{{{\text{d}}t }} & = k_{1} \cdot \left( {x_{3} \left( t \right) - x_{4} \left( t \right)} \right) \\ {\text{size}}\left( t \right) & = x_{1} \left( t \right) + x_{2} \left( t \right) + x_{3} \left( t \right) + x_{4} \left( t \right) \\ \end{aligned}$$

where *x*_1_ is the main cycling cells of the tumour and *x*_2_, *x*_3_ and *x*_4_ represent states of cell death following anticancer treatment. Cell growth in *x*_1_ is first exponential then followed by a linear growth phase, described by *λ*_0_ and *λ*_1_, respectively.Once the full data set was simulated, the tumour burden limit was applied, when an animal had an observed tumour size of 2.5 g or above that measurement and all subsequent measurements for that animal were excluded. The study was then truncated, as is often done in practice, with the final day chosen based on the number of animals in the control group, as when very few animals remain in the control group comparisons to the treated groups cannot be made.In this example, the comparison of dose groups is based on the estimation of the dose–response curve, where response is defined as tumour size on the final day of the study, as this allows a comparison with more traditional types of analysis. However, it is possible to use the resulting model to estimate drug efficacy in other ways. The true dose–response curve was simulated from the model and compared to the dose–response curve estimated using the average size from the simulated data following dropout. A *t* test was also carried out on the simulated data on the final day to see whether differences between the dose groups and the control could be detected.The four model-based methods described above were applied to the simulated data set. For each method, the same base model for tumour growth was used as the one used to simulate. All fitting was carried out in NONMEM 7.3 [18] using ADVAN13, example code for the censoring and joint modelling methods is included as an appendix. First-order conditional estimation (FOCE) with interaction was used for the modelling and pattern mixture methods, whilst Laplacian estimation was used for the censoring and joint modelling methods as in both methods continuous and categorical data are fitted simultaneously. Model diagnostics and visual predictive checks (VPC) were carried out on all models fitted throughout each method. From the resulting model, the dose–response curve was estimated using expected tumour size on the final day, with 95 % confidence intervals calculated from bootstrapping where possible.

### Real data

A real example was also analysed using the proposed methods. The data were from an AstraZeneca study into an anticancer compound (referred to as drug A), in combination with another drug (referred to as drug C), where a tumour burden limit of 2.5 g was used. The study included 48 animals in 6 equally sized groups, a control group, a 20 mg/kg dose of drug A group and a 10 mg/kg dose of drug C group, as well as three combination groups, where 5, 10 or 20 mg/kg of drug A was given, in addition to 10 mg/kg of drug C. Both drugs were given as single dose on day 13. The dose–response for drug A was considered in the four groups where 10 mg/kg of drug C was given.

*t* tests were carried out on the data at the latest time point where animals remained in each group to be compared. All the available data were then used to develop the model. The same base tumour growth model was used as in the simulated data example; however, a K-PD model was used in place of a PK model as no drug concentrations were available [19]. The PK was described using a “virtual” one-compartment model with bolus input, with the drug effect assumed to be proportional to a “virtual” infusion rate instead of drug concentration. In this example, as two drugs are being given, there are two dosing compartments, which are referred to as $x_{\text{Drug A}}$ and $x_{\text{Drug C}}$ as shown in Eq. 8. The drug concentrations in the tumour growth model in Eq. 7 were then replaced by DRA and DRC from Eq. 8. The two drugs were assumed to have an additive effect [20].

8 $$\begin{aligned} \frac{{{\text{d}}x_{\text{Drug A}} }}{{{\text{d}}t}} = - k_{{e,{\text{Drug A}}}} \cdot x_{\text{Drug A}} \left( t \right),\quad {\text{DRA}} = k_{{e,{\text{Drug A}}}} \cdot x_{\text{Drug A}} \hfill \\ \frac{{{\text{d}}x_{\text{Drug C}} }}{{{\text{d}}t}} = - k_{{e,{\text{Drug C}}}} \cdot x_{\text{Drug C}} \left( t \right) ,\quad {\text{DRC}} = k_{{e,{\text{Drug C}}}} \cdot x_{\text{Drug C}} \hfill \\ \end{aligned}$$

It should be noted that a proportion of the data in the treated groups was missing in the middle of the study as the tumours were too small to be measured; the use of the M3 method for this missing data was investigated. Animals were not assumed to be “cured” when a tumour was no longer measureable as they tended to regrow again before the end of the study [6].

## Results

### Simulated data

#### The data

**Fig. 2:**
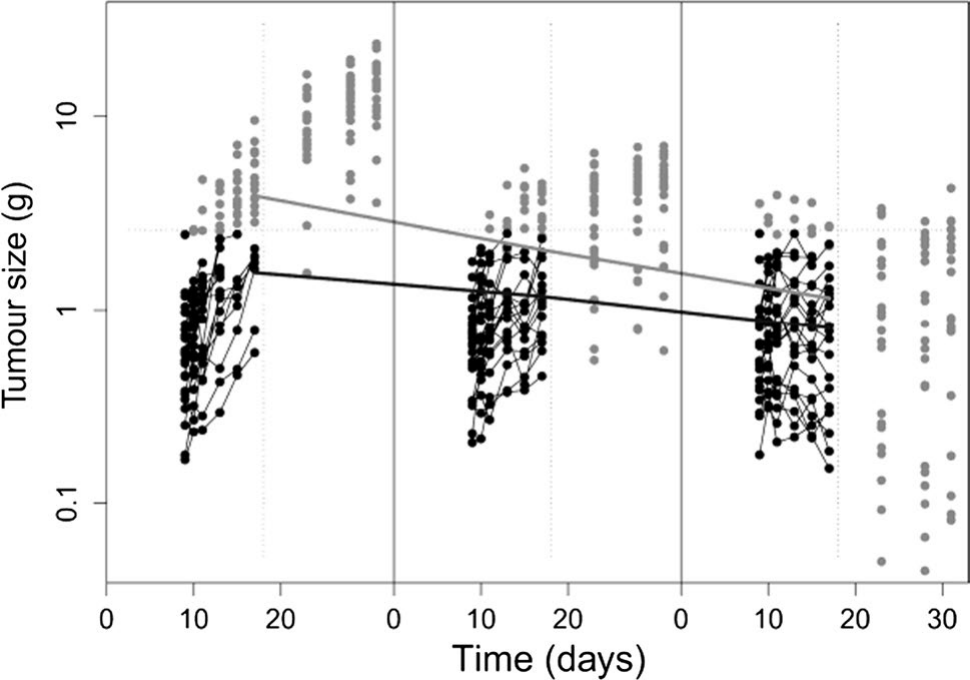
Simulated tumour growth profiles for control, 4 and 8 mg/kg groups, grey points were deleted as the animal had dropped out for being above the tumour burden limit or they occurred after 17 days, with those points in black making up the final data set. The *paler* *solid line* shows the average tumour sizes on the final day in each dose group before dropout occurred, with the *darker solid line* showing the average following dropout. The *dashed lines* show the cut-offs for TBL and end of study 69 × 58 mm (300 × 300 DPI)

The simulated data set is shown in Fig. 2, with observations missing due to dropout greyed out. The study was ended on day 17, as after this only one animal remained in the control group. It can be observed that the paclitaxel treatment is reducing tumour growth, and is also causing increased variation between individuals as the dose increases. The reduction in the average tumour size on the final day following dropout can be seen through the lines plotted through the points from day 17 on each plot before and after dropout. In total 82 of a possible 432 observations in the truncated study were missing (19 %), over half of which were from the control group, where in total 30 % of the data was missing. In the control group, only 8 animals completed the study, compared to 15 in the 4 mg/kg group and 19 in the 8 mg/kg group.

#### Overall results

The parameter estimates obtained using each of the methods are given in Table S1 in the supplementary material. In general, the parameter estimation is good, and all estimates are within 25 % of the true values, with the exception of the estimate of the linear growth phase (*λ*_1_) which is highly underestimated by the modelling and pattern mixture methods. In general, the inter-individual variation and residual error are less well estimated, the inter-individual variation (IIV) for *K*_1_ and *K*_2_ could not be well estimated together, so IIV on *K*_2_ was removed from the model for all analyses. The modelling method tended to overestimate the variation and underestimate the residual error, whilst the pattern mixture method overestimated both. The censoring and joint methods estimated the IIV well, but the residual error is inflated by both methods, particularly in the censoring method, which may be due to some model misspecification.

Where available, the relative standard errors (RSE) are reasonable for most of the parameter estimates, with the censoring method having the highest, and the joint model the lowest. However, some of the RSEs for the IIV estimates are high for the modelling and censoring methods, which were not observed when using the joint model, where all RSEs remained low. The modelling, censoring and joint modelling methods take a comparable amount of time. The pattern mixture method takes longer than the other methods as a model need to be fitted to each dropout pattern, and then to each of the imputed data sets, additional time is also required for the imputation of missing data and pooling of the results from each imputation.

The true dose–response can be assessed using the population tumour size at each dose, as simulated from the model, which can be seen in Table 1. The tumour sizes estimated using the average size on the final day are shown in the row below, and it can be seen that they provide a poor estimate, with the estimate in the control group particularly poor, making the dose–response curve nearly flat, and the drug appear less effective than it is. In general, as the tumour burden limit is reduced, the estimated dose–response curve will become lower and flatter. *t* test found that the tumour sizes in the high dose group were significantly smaller than those in the control group, but no difference was found between the lower dose group and the control group.

**Table 1:** Predicted tumour sizes on day 17 for each of the four methods, with 95 % confidence intervals from bootstrapping

Method	95 % CI around tumour size prediction on day 17		
	Control group	4 mg/kg group	8 mg/kg group
True values	4.90	1.91	0.68
Means	1.56 (1.00–1.88)	1.18 (0.93–1.49)	0.80 (0.60–1.11)
Modelling	3.19 (2.70–3.77)	1.68 (1.30–1.97)	0.68 (0.52–0.89)
Pattern mixture	2.40	1.36	0.62
Censoring	4.84 (3.38–7.84)	1.79 (1.19–2.91)	0.56 (0.43–1.12)
Joint modelling	5.22 (4.16–5.47)	1.99 (1.39–2.27)	0.64 (0.46–0.76)

The expected tumour sizes on day 17 from each method are also given in Table 1. The 95 % confidence intervals allow comparisons between the dose groups, such that if the intervals do not overlap the difference in tumour size can be considered significant at the 5 % level. This same information can be seen in the dose–response curves in Figure S1 in the supplementary material. The modelling method found a significant difference between both dose groups and the control group; however, the dose–response curve was not well estimated, with the response in the control group particularly poor and the true curve lying outside of the 95 % confidence interval. When using the censoring method, significant differences between the groups were found and the dose–response curve was well estimated but the confidence intervals were wide, mainly caused by the high residual error associated with this method. Finally, the joint model found that both groups were significantly different to the control group and the dose–response curve was well estimated, with confidence intervals much smaller than those found using the censoring method. The estimate of drug effect (*K*_2_) also provides an estimate of the drug’s effectiveness. It was underestimated in the modelling method and overestimated in the other two methods, which is consistent with the greater efficacy estimated by the second of the two methods.

The pattern mixture model may be affected by small sample size, as there are relatively few animals in each dropout pattern. For this reason, the dropout patterns were combined into four groups (A–D); dropout before day 11 with 7 animals, dropout on day 11 with 7 animals, dropout after day 11 with 16 animals and those who completed the study with 42 animals. This grouping meant there was a minimum of seven animals in each dropout pattern. The ACMV method was chosen, meaning the model fitted to animals in pattern A was used to impute missing values for animals in pattern B, and the model fitted to animals in patterns A and B was used to imputed missing values for animals in pattern C and so on.

**Fig. 3:**
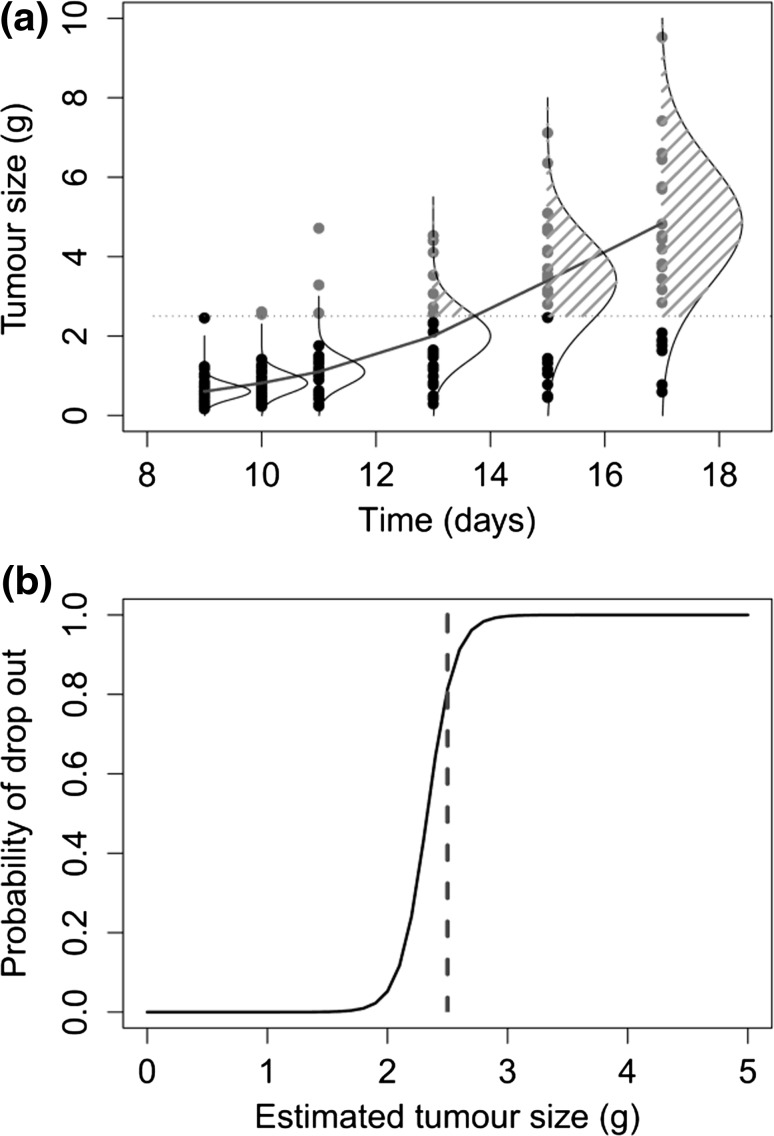
**a** Diagram explaining the use of the M3 method in the control group, with observed tumour sizes (*black*) and missing observations (*grey*), the population model (*solid line*), with the normal distribution around each predicted point. Adapted from Bonate’s book pharmacokinetic–pharmacodynamic modelling and simulation [21]. **b** Probability of dropout from logistic model of dropout by on tumour size 128 × 195 mm (300 × 300 DPI)

A plot of the M3 method for the control group is given in Fig. 3a, which shows the population model fit, with associated variation. In the M3 method, the likelihood for missing values is replaced by the probability and the tumour size was truly above the TBL given that the animal had been dropped from the study, which is represented by the shaded area under the normal curves. In this plot, it can be seen that the normal distributions capture the missing data (greyed out) well, despite the inflated residual error estimate.

The parameter estimates from the logistic dropout model of the joint modelling method were −20.4 for the intercept, 8.75 for the effect of tumour type, and the random effect CV % estimate was 11.3 %. This resulted in the probability of dropout curve in Fig. 3b, where the probability of dropout is close to zero until the tumour reaches approximately 2 g, then rises steeply, until, when the tumour reaches 3 g the probability of dropout is nearly 1.

### Real data

**Fig. 4:**
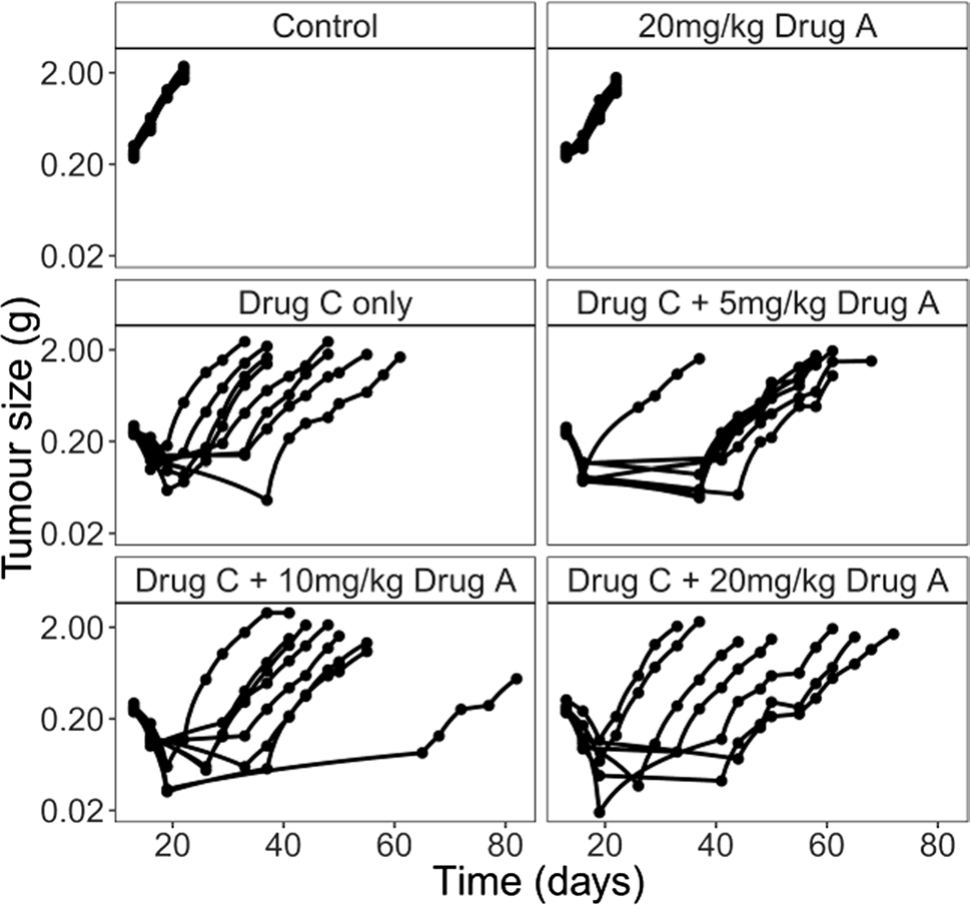
Tumour growth profiles from the real data by dose of drug A and drug C 77 × 72 mm (300 × 300 DPI)

The data used to estimate the dose–response of drug A are shown in Fig. 4. Across all dose levels, 16 animals dropped out before the end of the study, leading to 39 missing observations. The dose–response was estimated from the data using the average tumour size in each dose group on day 55 (Table 2). The tumour size is similar in both the drug C only group and the 10 mg/kg group and is lower in the 5 and 20 mg/kg groups, but the differences are not significant. Overall there is little evidence of drug A being efficacious, with the dose–response curve being relatively flat. These results will be dependent on the time point chosen for the analysis, and choosing an earlier time point may have led to drug A appearing more efficacious, as all treatments were single dose.

**Table 2:** Estimated tumour size on day 55 using each of the methods, with 95 % confidence intervals in parentheses calculated from bootstrapping

Method	95 % CI around tumour size prediction on day 55			
	Drug C	Drug C + 5 mg/kg drug A	Drug C + 10 mg/kg drug A	Drug C + 20 mg/kg drug A
Means	1.23 (0.69–1.78)	0.37 (0.23–0.63)	1.22 (1.09–1.35)	0.87 (0.68–1.06)
Modelling	3.18 (1.10, 4.25)	3.09 (0.92, 4.00)	3.00 (0.80, 3.92)	2.98 (0.52, 3.58)
Censoring	4.93 (3.04, 7.34)	4.37 (2.45, 6.52)	3.81 (1.85, 5.99)	2.70 (0.83, 5.01)
Joint modelling	4.20 (3.51, 5.07)	3.94 (3.24, 4.83)	3.68 (2.97, 4.58)	3.16 (2.42, 4.12)

There were 128 observations missing because the tumours were too small to measure. The number generally increased as the dose increased. Using the M3 method to account for these unmeasurable tumours was investigated but did not provide an improvement to the model. The parameter estimates from each of the methods are given in Table S2 in the supplementary material; the inter-individual variation could not be estimated for all parameters, due to the high number of parameters relative to the amount of data. The pattern mixture method could not be implemented due to the small number of animals, with only 16 animals dropping out altogether, there was a maximum of two animals in any dropout pattern, which was not enough to build a robust model for the imputation of missing data. In this example, the models were fitted starting at the time of first dose (day 13), not the time of inoculation (day 0).

The observed variation in the control and drug A only groups was very low, suggesting most of the variability is introduced by drug C; however, this could be due to the shorter follow-up time used in these groups (9 days compared to 69 days). As in the simulated example, the growth parameters (*λ*_0_ and *λ*_1_) are estimated to be higher in the censoring and joint modelling methods than in the modelling method, explaining the higher estimated tumour sizes on the final day. The standard errors follow a similar pattern to the simulated example, with the largest being observed for the censoring method, followed by the modelling method.

The estimated tumour size on day 55 in for each of the dose groups is given in Table 2, and the dose–response curves can be seen in Figure S2 in the supplementary material. When comparing the mean of the final measurements, the highest dose appears to have reduced the average size of the tumours by 0.4 g compared to the controls; the reduction is estimated to be even lower using the modelling methods at 0.2 g. However, when using the other two methods, the drug appears to be having a greater effect with the reduction estimated to be 2.2 g for the censoring method and 1 g for the joint modelling method. These findings are reflected in the estimate of the drug effect parameter ($K_{{2,{\text{Drug A}}}}$) which was estimated to be over ten times lower in the modelling method than the other two methods.

The results suggests that, as in the simulated example, the drug effect is larger than suggested by comparing final averages, as dropout could be hiding the treatment effect, by reducing the estimated tumour size in the control group, which can then be recovered by accounting for the dropout in the analysis.

## Discussion

Using any of the proposed methods to account for the dropout gives a better estimate of the dose–response than comparing the tumour sizes on the final day. The modelling and pattern mixture methods give the worst estimates mainly due to estimation of the tumour sizes in the control group, which remain underestimated. The pattern mixture model could also be struggling due to low sample size, here only 72 animals were available, leading to low numbers of animals in each dropout pattern, whereas another successful use of the method included data from over 1100 patients [12].

A partial reason for the poor performance of the modelling method is the underestimation of the linear growth phase. For the model used to simulate the data, it is expected that on average exponential growth will switch to linear growth at approximately 3 g, occurring around day 14 in the control group. This means very little data will remain to support the estimation of this linear growth phase, as animals drop out when their tumour reaches 2.5 g. However, it is believed that this does not fully account for the poor performance, and the initial exponential growth is also underestimated by the modelling method. Further examples were simulated from simpler one parameter growth models, and following dropout similar underestimation of tumour size was observed. As the pattern mixture method involved the fitting of the same model, it suffered from the same problems as modelling alone.

Both the censoring and joint modelling methods give good estimates of the dose–response. However, the censoring method gives poor estimates of the variation between animals and the residual error, meaning whilst it may be useful in giving a point estimate for the efficacy of the drug, it may not be suitable for future simulations. The between-animal variation is well estimated by the joint model, making it the most effective all-around method for accounting for dropout due to the tumour burden limit. Joint modelling also allows some flexibility around the tumour burden limit, which may be important in practice, although not in the artificial simulated example discussed here. Both methods are relatively easy to implement, and analysis takes no longer than modelling alone.

The modelling, censoring and joint modelling methods could all detect differences between both the low and high dose groups and the control group, which was not possible when using a *t* test, which may mean fewer animals could be used in order to get the same results using one of these methods, in keeping with both the reduction and refinement of the 3Rs principles [22].

The success of the methods was assessed through the dose–response curve, where response was defined as the estimated tumour size on the final day; this was done so that a comparison could be made to other methods that compare the tumour sizes on the final day. However, this may not be the best way to assess the differences between groups. Other metrics could be used, such as the parameter describing the drug effect (*K*_2_) in the above cases, or area under the tumour growth inhibition curve, which may be more effective at summarising the tumour growth throughout the whole experiment.

The simulation study shows that the joint model is the most effective of the proposed methods, with the dose–response, and variation between animals well estimated and the method easy to implement. The real case example shows the joint modelling method can help to recover the estimate of drug effect when it has been disguised by dropout.

### Electronic supplementary material

Below is the link to the electronic supplementary material.

Supplementary material 1 (PDF 486 kb)

## Appendix: NONMEM code

### NONMEM code to implement the joint modelling method

As in the censoring method except:

## References

[1] Tan M, Fang HB, Tian GL, Houghton PJ (2005). Repeated-measures models with constrained parameters for incomplete data in tumour xenograft experiments. Stat Med.

[2] Hather G, Liu R, Bandi S, Mettetal J, Manfredi M, Shyu WC, Donelan J, Chakravarty A (2014). Growth rate analysis and efficient experimental design for tumor xenograft studies. Cancer Inform.

[3] Houghton PJ, Morton CL, Tucker C, Payne D, Favours E, Cole C, Gorlick R, Kolb EA, Zhang W, Lock R, Carol H, Tajbakhsh M, Reynolds CP, Maris JM, Courtright J, Keir ST, Friedman HS, Stopford C, Zeidner J, Wu J, Liu T, Billups CA, Khan J, Ansher S, Zhang J, Smith MA (2007). The pediatric preclinical testing program: description of models and early testing results. Pediatr Blood Cancer.

[4] Hogan JW, Laird NM (1997). Mixture models for the joint distribution of repeated measures and event times. Stat Med.

[5] Workman P, Aboagye EO, Balkwill F, Balmain A, Bruder G, Chaplin DJ, Double JA, Everitt J, Farningham DA, Glennie MJ, Kelland LR, Robinson V, Stratford IJ, Tozer GM, Watson S, Wedge SR, Eccles SA, Committee of the National Cancer Research I (2010). Guidelines for the welfare and use of animals in cancer research. Br J Cancer.

[6] Pan JX, Bao YC, Dai HS, Fang HB (2014). Joint longitudinal and survival-cure models in tumour xenograft experiments. Stat Med.

[7] Mould DR, Walz AC, Lave T, Gibbs JP, Frame B (2015). Developing exposure/response models for anticancer drug treatment: special considerations. CPT Pharmacomet Syst Pharmacol.

[8] Rubin DB (1976). Inference and missing data. Biometrika.

[9] Diggle P, Kenward MG (1994). Informative drop-out in longitudinal data-analysis. J R Stat Soc C Appl.

[10] Wu MC, Carroll RJ (1988). Estimation and comparison of changes in the presence of informative right censoring by modeling the censoring process. Biometrics.

[11] Bjornsson MA, Friberg LE, Simonsson US (2015). Performance of nonlinear mixed effects models in the presence of informative dropout. AAPS J.

[12] Yuen E, Gueorguieva I, Aarons L (2014). Handling missing data in a duloxetine population pharmacokinetic/pharmacodynamic model—imputation methods and selection models. Pharm Res.

[13] Hu CP, Sale ME (2003). A joint model for nonlinear longitudinal data with informative dropout. J Pharmacokinet Pharmacodyn.

[14] Hansson EK, Amantea MA, Westwood P, Milligan PA, Houk BE, French J, Karlsson MO, Friberg LE (2013). PKPD modeling of VEGF, sVEGFR-2, sVEGFR-3, and sKIT as predictors of tumor dynamics and overall survival following sunitinib treatment in GIST. CPT Pharmacomet Syst Pharmacol.

[15] Simeoni M, Magni P, Cammia C, De Nicolao G, Croci V, Pesenti E, Germani M, Poggesi I, Rocchetti M (2004). Predictive pharmacokinetic-pharmacodynamic modeling of tumor growth kinetics in xenograft models after administration of anticancer agents. Cancer Res.

[16] Rubin DB (1987). Multiple imputation for nonresponse in surveys.

[17] Beal SL (2001). Ways to fit a PK model with some data below the quantification limit. J Pharmacokinet Pharmacodyn.

[18] Beal S, Sheiner LB, Boeckmann A, Bauer RJ (2009) NONMEM User’s guides (1989–2009)

[19] Jacqmin P, Snoeck E, van Schaick EA, Gieschke R, Pillai P, Steimer JL, Girard P (2007). Modelling response time profiles in the absence of drug concentrations: definition and performance evaluation of the K-PD model. J Pharmacokinet Pharmacodyn.

[20] Koch G, Walz A, Lahu G, Schropp J (2009). Modeling of tumor growth and anticancer effects of combination therapy. J Pharmacokinet Pharmacodyn.

[21] Bonate PL (2005). Pharmacokinetic-pharmacodynamic modeling and simulation.

[22] Russell WMS, Burch RL (1959) The principles of humane experimental technique. Methuen. http://worldcat.org. http://catalog.hathitrust.org/api/volumes/oclc/1486173.html

