# Supplementary material for: Accounting for dropout in xenografted tumour efficacy studies: integrated endpoint analysis, reduced bias and better use of animals
Source: Cancer Chemother Pharmacol. 2016 May 25;78:131–41. doi: 10.1007/s00280-016-3059-x (PMC4921113; doi:10.1007/s00280-016-3059-x)
Supplement: Supplementary file 1 — Supplementary material 1 (PDF 486 kb) [file 280_2016_3059_MOESM1_ESM.pdf]

Supplementary material for the manuscript "Accounting for dropout in xenografted tumour efficacy studies: Integrated endpoint analysis, reduced bias and better use of animals" submitted to the journal

Cancer Chemotherapy and Pharmacology

E. Martin[1], L. Aarons[1], W.T. Yates[2]

[1] Centre for Applied Pharmacokinetic Research, Manchester Pharmacy School, the University of Manchester, M13 9PT, U.K

[2] AstraZeneca, Innovative Medicines, Oncology, Modelling and Simulation, Li Ka Shing Centre, Robinson Way, Cambridge CB2 0RE, U.K

Corresponding author:
Emma Martin
E-mail: emma.martin@manchester.ac.uk

	True value	Modelling	Pattern mixture	Censoring	Joint modelling
**Parameter estimates**					
K_1 (1/day)	0.968	0.848 (27)	0.883	0.718 (34)	0.968 (6)
K_2 (L/mg.day)	0.629	0.582 (9)	0.552	0.790 (9)	0.694 (3)
λ_0 (1/day)	0.273	0.249 (8)	0.252	0.299 (6)	0.283 (2)
λ_1 (g/day)	0.814	0.384 (16)	0.229	0.723 (38)	0.729 (4)
$Size_0$ (g)	0.055	0.0654 (18)	0.0649	0.0410 (60)	0.0540 (4)
**Inter-individual variation estimates (CV%)**					
k1	14.5	27.0 (200)	49.0	18.9 (337)	13.2 (6)
k2	8.03	-	-	-	-
λ0	8.08	23.1 (28)	28.0	5.7 (521)	6.3 (10)
λ 1	2.3	12.0 (275)	29.9	3.3 (121)	3.4 (8)
$Size_0$	31.6	21.2 (127)	39.5	28.4 (302)	32.4 (8)
**Proportional error estimate (%)**					
	5.00	4.15 (7)	9.33	31.8 (0.1)	7.35 (7)
**Time taken (minutes)**					
	-	26.5	~ 128*	27.6	16.5

Table S1 Tumour growth model parameter estimates from each of the four methods. Relative standard errors from bootstrapping are given in parentheses. * refers to the time taken to fit the 8 models required to implement the pattern mixture model

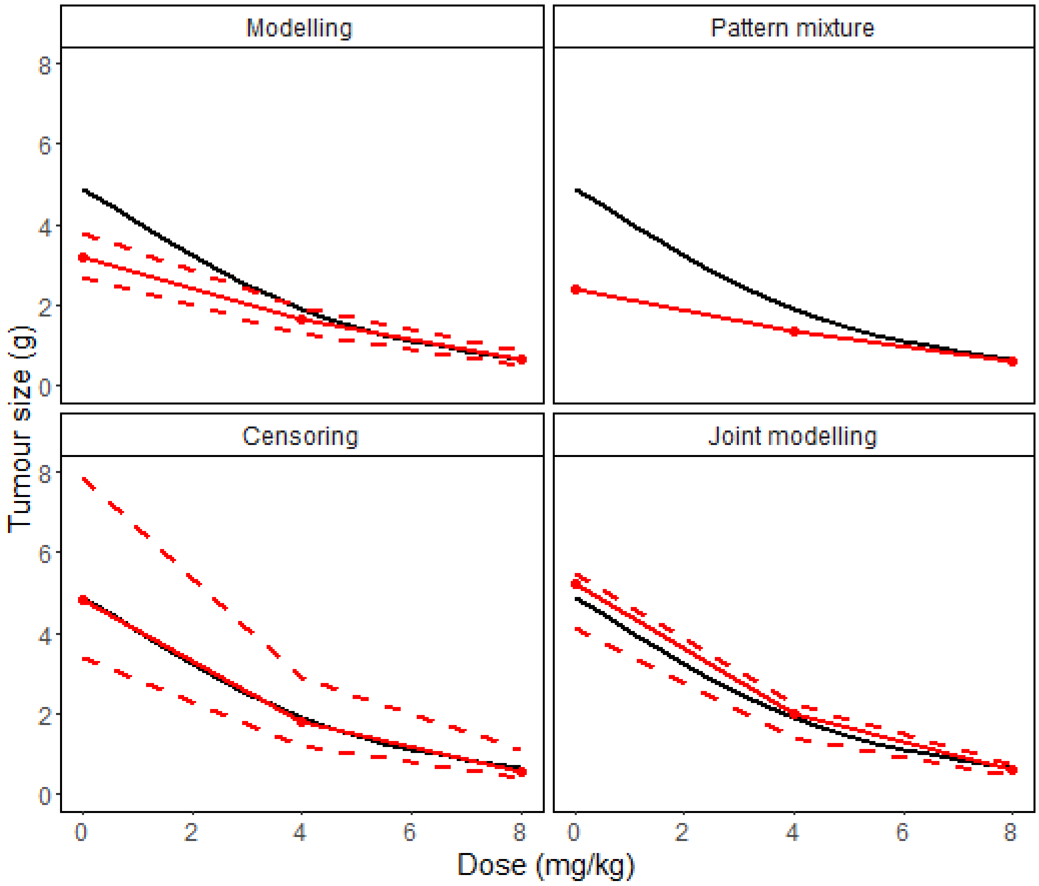

Figure S1 Dose response curves (solid red line) for the four methods applied to the simulated data set with 95% confidence intervals (dashed lines) from bootstrapping, compared to the true dose response curve (black)

	Modelling	Censoring	Joint modelling
**Kinetic parameters estimates**			
$Ke_{Drug\ A}$ (mg/kg.day)	0.459 (390)	0.504 (38)	0.622 (4)
$Ke_{Drug\ C}$ (mg/kg.day)	18.5 (14)	18.8 (10)	17.1 (5)
**Tumour growth model parameters**			
K_1 (1/day)	0.523 (13)	0.429 (31)	0.505 (9)
$K_{2,Drug\ A}$ (L/mg.day)	0.00593 (155)	0.105 (32)	0.0966 (3)
$K_{2,Drug\ C}$ (L/mg.day)	3.70 (7)	4.19 (26)	4.29 (6)
λ_0 (1/day)	0.150 (22)	0.190 (25)	0.270 (1)
λ_1 (g/day)	0.122 (17)	0.203 (18)	0.196 (6)
$Size_0$ (g)	0.210 (5)	0.202 (6)	0.175 (6)
**Inter-individual variation estimates (CV%)**			
$Ke_{Drug\ C}$	29.7 (62)	36.7 (324)	8.6 (21)
K_1	8.6 (85)	9.8 (78)	8.1 (29)
$K_{2,Drug\ C}$	3.7 (558)	31.4 (164)	13.9 (1)
λ_0	47.4 (42)	10.0 (134)	8.8 (23)
λ_1	11.6 (321)	11.4 (360)	9.1 (12)
**Proportional error estimate (%)**			
	8.93 (35)	38.1 (26)	6.02 (146)

Table S2 Parameter estimates from the Simeoni model fitted to the real data. Relative standard errors from bootstrapping are given in parentheses.

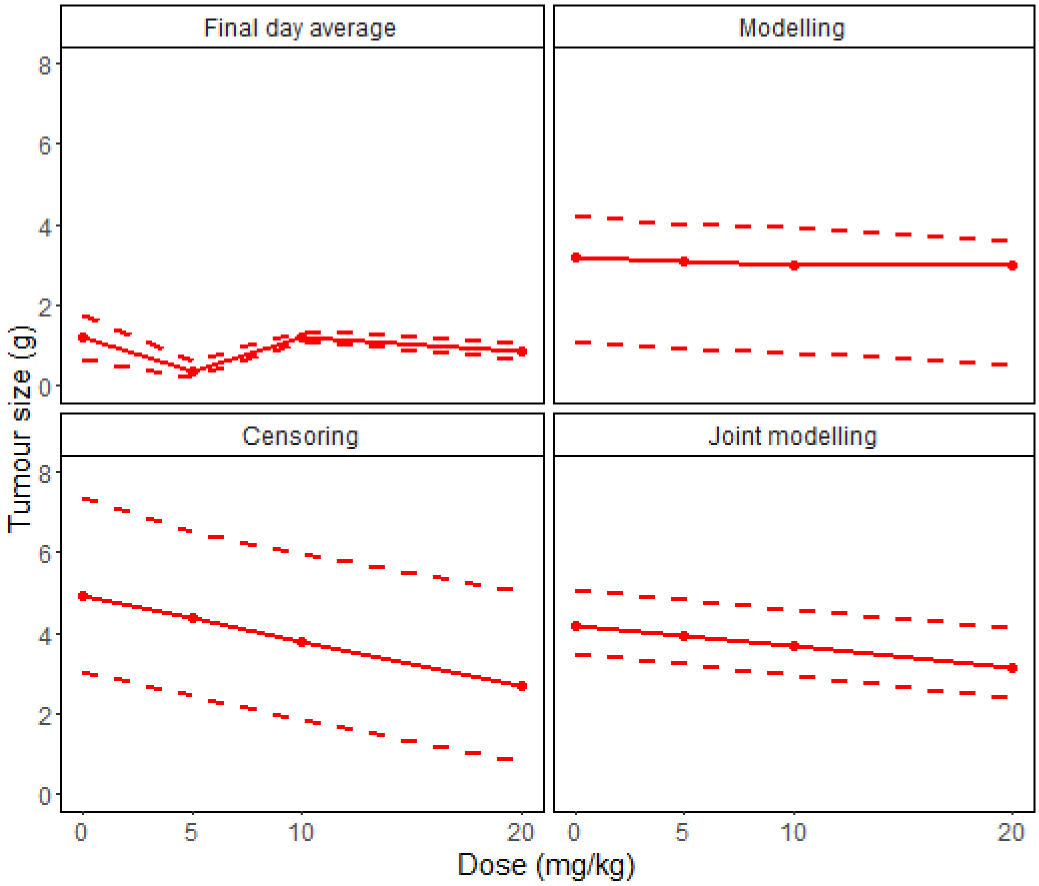

Figure S2 Dose response curves (solid red line) for the method of taking averages on the final day of the study, and the three methods accounting for dropout used in the real example, with 95% confidence intervals (dashed lines) from bootstrapping
